# Patient Preferences and Willingness to Pay for Cervical Cancer Prevention in Zambia: Protocol for a Multi-Cohort Discrete Choice Experiment

**DOI:** 10.2196/10429

**Published:** 2018-07-25

**Authors:** Sujha Subramanian, Yevgeniya Kaganova, Yuying Zhang, Sonja Hoover, Namakau Nyambe, Leeya Pinder, Carla Chibwesha, Sharon Kapambwe, Groesbeck Parham

**Affiliations:** ^1^ RTI International Waltham, MA United States; ^2^ University of North Carolina Lusaka Zambia; ^3^ University Teaching Hospital Lusaka Zambia; ^4^ University of North Carolina Chapel Hill, NC United States; ^5^ Ministry of Health Zambia Zambia; ^6^ University of Zambia Lusaka Zambia

**Keywords:** discrete choice experiment, cervical cancer prevention, economic evaluation

## Abstract

**Background:**

Although most countries in southern Africa have cervical cancer screening programs, these programs generally fail to reach a significant majority of women because they are often implemented as pilot or research projects, and this limits their scope and ability to scale up screening. Some countries have planned larger-scale programs, but these have either never been implemented or have not been successfully scaled up. Most of the global burden of cervical cancer is experienced in countries with limited resources, and mortality from cervical cancer is the most common cause of cancer-related deaths among women in Sub-Saharan Africa.

**Objective:**

The purpose of this study is to learn about preferences for cervical cancer screening in Zambia, to identify barriers and facilitators for screening uptake, and to evaluate willingness to pay for screening services to support the scaling up of cervical cancer screening programs.

**Methods:**

We will conduct a discrete choice experiment by interviewing women and men and asking them to choose among constructed scenarios with varying combinations of attributes relevant to cervical cancer screening. To inform the discrete choice experiment, we will conduct focus groups and interviews about general knowledge and attitudes about cervical screening, perception about the availability of screening, stigma associated with cancer and HIV, and payment for health care services. For the discrete choice experiment, we will have a maximum design of 120 choice sets divided into 15 sets of 8 tasks each with a sample size of 320-400 respondents. We will use a hierarchical Bayesian estimation procedure to assess attributes at the following two levels: group and individual levels.

**Results:**

The model will generate preferences for attributes to assess the most important features and allow for the assessment of differences among cohorts. We will conduct policy simulations reflecting potential changes in the attributes of the screening facilities and calculate the projected changes in preference for choosing to undergo cervical cancer screening. The findings from the discrete choice experiment will be supplemented with interviews, focus groups, and patient surveys to ensure a comprehensive and context-based interpretation of the results.

**Conclusions:**

Because willingness to pay for cervical cancer screening has not been previously assessed, this will be a unique and important contribution to the literature. This study will take into account the high HIV prevalence in Sub-Saharan Africa and prevailing gender attitudes to identify an optimal package of interventions to reduce cervical cancer incidence. This simulation of women’s decisions (and men’s support) to undergo screening will lay the foundation for understanding the stated preferences and willingness to pay to help design future screening programs.

**Registered Report Identifier:**

RR1-10.2196/10429

## Introduction

Around the world, a woman dies of cervical cancer about every 2 minutes [1], with 528,000 new cases and 266,000 deaths in 2012. Cervical cancer affects women during their most productive years because the majority are diagnosed under the age of 50; therefore, cervical cancer adversely affects not only women but also their families [2]. A large proportion of the global burden is experienced in countries with limited resources, and mortality from cervical cancer is the most common cause of cancer-related deaths among women in Sub-Saharan Africa [3,4]. The high prevalence of HIV in Sub-Saharan Africa, the focal point of the HIV/AIDS epidemic, makes the cervical cancer burden even more acute in these countries because women with HIV have a much higher incidence rate of cervical cancer than uninfected women [5]. Fortunately, given the advances in HIV/AIDS treatment in Sub-Saharan Africa, women are living longer with HIV, but unfortunately, they are dying from cervical cancer due to the scarcity of large-scale implementation of screening programs.

Cervical cancer is preventable, and early diagnosis is possible using low-cost technologies [6]. The World Health Organization guidelines recommend several screening approaches, including tests for human papillomavirus (HPV) and cytology (Pap test) and visual inspection with acetic acid (VIA) [7]. The screen-and-treat approach using VIA or low-cost rapid HPV test is the favored approach in the limited resource setting because it minimizes loss to follow-up [8]. Therefore, the knowledge and technology base exists to prevent and screen for cervical cancer in low-resource settings and, as in other settings, large-scale sustainable screening programs can be implemented [9-11].

Although most countries in southern Africa have a cervical cancer screening program that is either administered by the government or nongovernmental organizations, these programs generally fail to reach a significant majority of women [12]. This is largely because most screening activities in southern Africa are part of pilot or research projects, which limits their scope and ability to scale up screening. Some countries have planned larger-scale programs, but these have either never been implemented or have not been successfully scaled up. For example, Malawi attempted a nationwide cytology-based cervical cancer screening program, but it quickly deteriorated owing to lack of resources, trained professionals, and infrastructure [13].

Therefore, there is an urgent need to implement low-cost approaches already available for the prevention and early detection of cervical cancer in Sub-Saharan Africa. However, to date, no study has systematically evaluated preferences for prevention and screening, and this information is required to design and implement programs that will result in optimal uptake. Additionally, the financing of prevention and screening services is a significant barrier for scale-up.

Although discrete choice experiments (DCEs) have been performed in the context of maternal and child health, health infrastructure, and workforce development in Africa [14-16], no study has used validated quantitative methods to evaluate the willingness to pay for cervical cancer screening in Africa. Fee contributions based on an individual’s ability to pay, with safeguards for ensuring free access for the disadvantaged population, can provide a continuous, even if small, stream of revenue to allow for the sustainability of program operations. In this study, we will use DCE to elicit preferences for cervical cancer screening to identify barriers and facilitators for screening uptake and evaluate the willingness to pay for screening services, which can inform innovative financing arrangements to ensure sustainability. This study will be conducted in Zambia, one of the countries with the highest burden of cervical cancer in Sub-Saharan Africa.

## Methods

### Framework for Designing and Conducting the Discrete Choice Experiments

**Figure 1:**
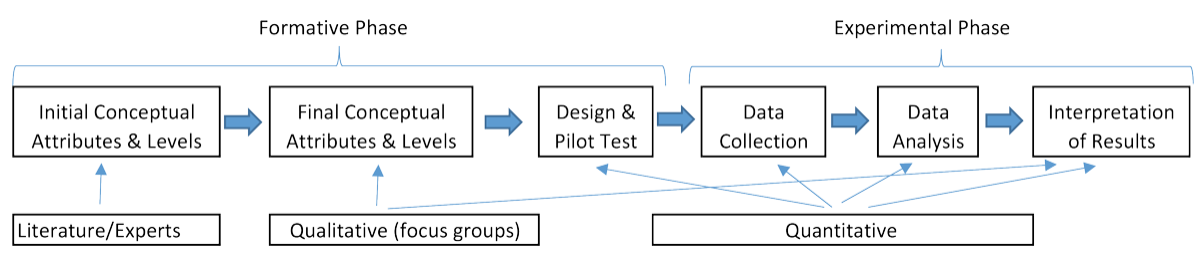
Mixed-methods framework for the discrete choice experiment.

In this study, women and men or partners of eligible women will be asked to choose among constructed scenarios with varying combinations of key attributes relevant to cervical cancer screening (for example, type of provider, cost, and distance to facility); choosing to have no screening will also be an option. The DCE approach is preferred over asking women and men about their willingness to pay directly in surveys or interviews [17-19]. DCE allows participants to choose among scenario combinations, an approach which provides them with a more natural consumer choice experience. Figure 1 provides an overview of the mixed-methods approach for implementing DCE. We will begin by identifying initial concepts and attributing levels for DCE based on feedback from experts and a review of the literature related to cervical cancer screening barriers and facilitators. Next, we will conduct a series of focus groups and interviews with stakeholders in Zambia to finalize the attributes and levels.

### Focus Groups and In-Depth Interviews

We will conduct two sets of focus groups with women and interviews with a range of stakeholders including women and men who are between the ages of 25 and 49 years. We plan to interview at least 8 individuals in each of the following groups: women who are HIV-negative, those who are HIV-positive, those who have been diagnosed with precancerous lesions, and those treated for cancer.

We will also interview men in both urban and rural locations. The focus groups and interviews will not address personal issues but will be directed at understanding general knowledge and attitudes about cervical screening, perception about the availability of screening, stigma associated with cancer and HIV, and payment for health care services. Written consent will be obtained from all participants, and the consent form will be translated into the local languages of Bemba, Nyanja, and Tonga. We will also interview providers to gain knowledge about the delivery of health care services, the use of cervical cancer screenings, and facilitators and barriers related to cervical cancer screening, diagnosis, and treatment. The key objective of these discussions is to gain insight to finalize the attributes and corresponding levels for DCE.

We will use a structured data collection template for standardization and coding to facilitate analysis. We will combine the qualitative information from all stakeholders to assess convergence around common themes and identify potential differences in viewpoints expressed by participants on specific topics. The prespecified coding scheme (refined as needed) will be used to perform a comparative assessment of barriers and facilitators, program attributes, and financing options. To assist in interpreting the findings, we will visually display the data in tabular and graphical formats.

### Discrete Choice Experiments Graphics, Choice Set Selection, and Supplementary Survey

**Figure 2:**
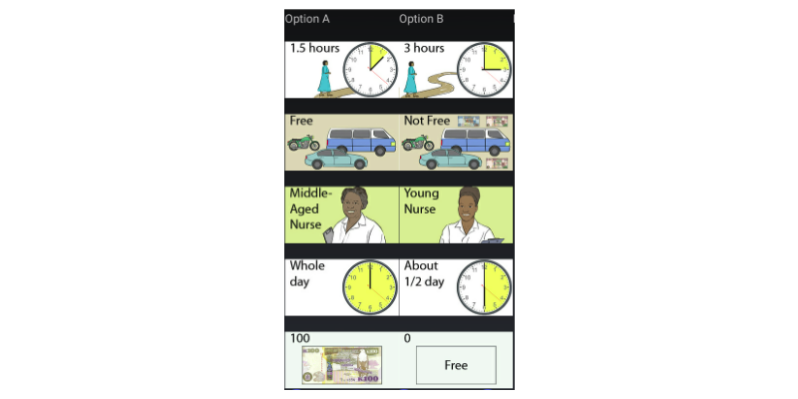
Discrete choice experiment: example graphics.

To permit use in a low-literacy environment, the attributes and levels will be converted into graphics (see example of the attributes and levels and the graphics provided in Table 1 and Figure 2). Our DCE selection (Table 1) will generate 200 possible scenarios (5^2^×2^3^), which are too many for any single respondent to assess. To select a fractional set of scenarios, we will employ a randomized design process that ensures that respondents see well-balanced and near-orthogonal fractions of the full-choice design (8 choice decisions per respondent). We will have no more than 128 profiles that will need to be tested after developing the fractional set of scenarios. Wong et al reported that even 729 possible profiles can be reduced to 128 profiles with a fractional factorial design [20]. In addition to the choice tasks, we will administer a supplemental survey to obtain demographics, socioeconomic status, reproductive history (women only), cervical cancer screening knowledge and use (women only), HIV status, access to care, attitudes toward using formal medical care (compared with traditional medicine), and stigma associated with HIV and cancer diagnosis.

### Cohort Selection and Sample Size

A key design consideration for DCE is to allow for both group-level and individual-level differences. The key groups of interest are HIV-positive women, HIV-negative women, women with unknown HIV status, and men (male partners). In addition, it is important to distinguish between urban and rural cohorts because they can differ in terms of participant attitudes, cultural sensitivities, and health care infrastructure. Finally, to also capture health-seeking behavior and potential underlying differences between those who attend health clinics and those who do not (owing to stigma, religion, traditional beliefs, cost, and other reasons) [21], we also will draw specific cohorts from health clinics and another set of cohorts from the wider community. We describe the 7 cohorts targeted for DCE in Textbox 1.

Sample size calculation for DCE studies in health care is an evolving field. Johnson et al simulated sample sizes to estimate precision that could be obtained for DCE studies [22]. They found that precision increases rapidly at lower sample sizes (less than 150 observations) and then flattens out at around 300 observations. Based on this, the rule of thumb for DCEs is that generally 300-400 cases per group are adequate. Hall et al and Lancsar and Louviere have indicated that about 20-25 respondents per choice set can provide precise parameter estimates [23,24]. Our proposed study, with a maximum design of 120 choice sets that will be divided into 15 sets of 8 tasks each, can achieve this with a sample size of 320 to 400. Another approach recommended by Johnson and Orme [25] suggests that the sample size required for the main effects depends on the number of choice tasks (*t*), the number of alternatives (*a*), and the number of analysis cells (*c*) according to the following equation: N>500*c*/(*t*×*a*). When considering the main effects, *c* is equal to the largest number of levels for any of the attributes. For our proposed model, the values are *t*=8, *a*=2 (without option to select neither choice), and *c*=5 (main effects) or *c*=10 (based on planned interaction between 5-level and 2-level attributes); therefore, for the main effects, N can be estimated as (500×5)/(8×2)=156.25 observations and for effects with interactions, N can be estimated as (500×10)/(8×2)=312.50 observations.

**Table 1:** Attributes and levels for the discrete choice experiment.

Attribute	Level
Distance (hours by foot)	1, 1½, 2, 2½, 3
Transport	Free; not free
Provider	Middle-aged nurse; young nurse
Wait time	About half a day; full day
Cost (in Zambian kwacha)	0, 25, 50, 75, 100

**Study cohorts for the discrete choice experiment. For each of the 7 groups, 400 individuals were selected. A previously conducted pilot test included 15 women and 15 men.**

1. **Health clinic cohort**
  - HIV-positive women
  - HIV-negative and unknown women
  - Men (male partners of women when possible)
2. **Community cohort**
  - Urban women
  - Urban men
  - Rural women
  - Rural men
3. **Justification for cohort selection**
  - Health clinic: To systematically identify HIV-positive women; women who attend clinics may also differ from the general population (owing to stigma, traditional beliefs, cost, etc).
  - Community: To understand screening preferences from cohorts that are most likely to reflect the general population.

In all the approaches identified above, a sample size of 400 per group will be adequate to perform DCE to obtain preferences and willingness to pay estimates for each of the 7 targeted cohorts. Therefore, we will include a total sample of 2800 individuals.

### Identifying Discrete Choice Experiments Participants

Two health clinics that treat middle- and low-income individuals in the Lusaka area will be randomly selected for the study; the government operates multiple community-based clinics that offer a similar set of services to our target cohort and, therefore, we will be able to select a representative group of participants. The study interviewers (recruited based on experience performing similar studies) will visit the health centers to recruit participants on a continuous basis by inviting eligible women and men to complete the survey. Written consent will be obtained, and the survey will be conducted in an allocated room at the health center. For the community cohorts, we will identify respondents in Lusaka (urban) and appropriate rural or semirural setting. The interviews will take place at the residence of the participant or at a nearby community center.

### Discrete Choice Experiments Data Collection Process

We will train a team of 4 interviewers (fluent in English and one of the other local languages) and a supervisor, who will be responsible for the data collection. The graphics and software created by RTI International (Research Triangle Park, North Carolina, USA) will be loaded onto tablets to allow easy manipulation and viewing by the respondent. The supplemental survey and data collection pertaining to the DCE choices will be entered directly into the tablet by the interviewer with quality control features to ensure accuracy of data input formats (dates, ages, and completeness of responses to questions). We will pilot-test the data collection process with 30 women and men (15 each) selected to reflect the targeted cohorts from Lusaka and rural districts. We will introduce all respondents to the graphics by presenting each illustration separately and explaining the attribute and level in detail. Each participant will become acquainted with the DCE approach through three warm-up example choice decisions prior to the presentation of the selected tradeoffs for that participant. After presenting the DCE choices, the interviewer will verbally pose the survey questions and record answers in the preformatted data collection tool (approximately 45 minutes will be required for the interviews). Respondents will be interviewed by one of the 4 trained interviewers or coordinator or supervisor as needed. The data will be reviewed on a daily basis by the supervisor for quality control (so that any issues identified can be quickly rectified) and uploaded as soon as possible into a central deidentified database that will be password-protected.

### Discrete Choice Experiments Data Analysis and Interpretation

A hierarchical Bayesian estimation procedure will be used to assess attributes at the group and individual levels. Hierarchical regressions simultaneously assess relationships within a given level and between or across levels. This technique allows for independent variance to be calculated for both levels simultaneously. The model will generate preferences for attributes to assess the most important features and also allow for the assessment of differences among cohorts (eg, HIV-positive women vs others). We will conduct policy simulations reflecting potential changes in the attributes of the screening facilities and calculate the projected changes in preference for selecting to undergo cervical cancer screening. The findings from DCE will be supplemented with information gained from other qualitative (interviews and focus groups) and quantitative (patient supplemental survey) data collection to ensure a comprehensive and context-based interpretation of the results.

This study has received ethical approval from the Institutional Review Boards at RTI International and the University of Zambia’s Biomedical Research Ethics Committee. In addition, the Zambian Ministry of Health has reviewed and approved this study.

### Availability of Data and Material

We will abide by National Institutes of Health policies and make the data from this study available to other researchers.

## Results

The project was funded in July 2016 and ethical approval was obtained for the discrete choice experiment in April 2017. Enrolment is currently ongoing and we plan to complete data collection by August 2018. First results are expected to be submitted for publication in 2019.

## Discussion

Because willingness to pay for cervical cancer screening has not been previously assessed, this will be a unique and important contribution to the literature. Sub-Saharan African countries have faced challenges in scaling up cervical cancer screening, and the ability to finance these programs has been one key barrier. This study, by addressing stakeholder preferences across key stakeholders as well as HIV-negative and -positive women and men, will take into account the high HIV prevalence in Sub-Saharan Africa and prevailing gender attitudes to identify an optimal package of interventions to reduce cervical cancer incidence.

Although DCE is a useful approach to elicit tradeoffs and choices, this experiment may not be able to account for all the contextual and institutional factors that affect actual behavior, especially given the complex nature of health care decision making. We will use the additional qualitative and quantitative information collected during the DCE implementation and incorporate background contextual aspects in reaching conclusions based on the DCE results. Despite the potential limitations of DCEs, this simulation of women’s decisions (and men’s support) to undergo screening will lay the foundation for understanding the stated preferences and willingness to pay to help design future screening programs. A more systematic national implementation process that is evidence-based, data-driven, and resource-based is required for long-term sustainable cancer control. The establishment of screening programs in high income countries have resulted in dramatic decreases in the incidence of cervical cancer [10,11], and with better implementation of tailored programs in Sub-Saharan, a similar decline can be achieved.

## Appendix

Multimedia Appendix 1 Peer-reviewer report.

## Abbreviations

DCE: discrete choice experiment
VIA: visual inspection with acetic acid
HPV: human papillomavirus
