# Supplementary material for: Patient Preferences and Willingness to Pay for Cervical Cancer Prevention in Zambia: Protocol for a Multi-Cohort Discrete Choice Experiment
Source: JMIR Res Protoc. 2018 Jul 25;7(7):e10429. doi: 10.2196/10429 (PMC6083044; doi:10.2196/10429)
Supplement: Multimedia Appendix 1 [file resprot_v7i7e10429_app1.pdf]

SUMMARY STATEMENT
(Privileged Communication)

PROGRAM CONTACT:
Sarah Kobrin
(240) 276-6931
kobrins@mail.nih.gov

Release Date: 10/27/2015

Application Number: 1 R01 CA200845-01A1

Principal Investigator

SUBRAMANIAN, SUJHA PHD

Applicant Organization: RESEARCH TRIANGLE INSTITUTE

Review Group: DIRH
Dissemination and Implementation Research in Health Study Section

Meeting Date: 10/14/2015
Council: JAN 2016
Requested Start: 04/01/2016
RFA/PA: PAR13-055
PCC: B9SC
Dual IC(s): AI

Project Title: Evidence-based Approaches, Financing and Tools for Cost-Effective Scale-up Cervical Cancer Prevention in Sub-Saharan Africa
SRG Action: Impact Score: 29 Percentile: 17
Next Steps: Visit http://grants.nih.gov/grants/next_steps.htm
Human Subjects: 30-Human subjects involved - Certified, no SRG concerns
Animal Subjects: 10-No live vertebrate animals involved for competing appl.
Gender: 1A-Both genders, scientifically acceptable
Minority: 5A-Only foreign subjects, scientifically acceptable
Children: 3A-No children included, scientifically acceptable
Clinical Research - not NIH-defined Phase III Trial

Project Year	Direct Costs Requested	Estimated Total Cost
1	329,150	520,669
2	448,927	710,139
3	338,569	535,569
TOTAL	1,116,646	1,766,377

ADMINISTRATIVE BUDGET NOTE: The budget shown is the requested budget and has not been adjusted to reflect any recommendations made by reviewers. If an award is planned, the costs will be calculated by Institute grants management staff based on the recommendations outlined below in the COMMITTEE BUDGET RECOMMENDATIONS section.

EARLY STAGE INVESTIGATOR, NEW INVESTIGATOR

1R01CA200845-01A1 SUBRAMANIAN, SUJHA

EARLY_STAGE_INVESTIGATOR
NEW INVESTIGATOR

RESUME AND SUMMARY OF DISCUSSION: The application proposes a systematic process evaluation of the Cervical Cancer Prevention Program in Zambia (CCPPZ) to identify lessons for scaling up services in other sub-Saharan countries. Investigators will undertake an assessment of stakeholder perspectives on current program operations to identify barriers and facilitators and financing arrangements to ensure sustainability, and develop a microsimulation model. The application has many strengths including the significant public health issue addressed in a context of HIV/HPV infection and need for policy-related information. The innovative development of a dynamic model incorporating real world data, and incorporation of REAIM bode well for success. The team is strong and has addressed the prior critiques well. However, a number of concerns remain. These are mainly within the cost study, which lacks important components such as details about user fee arrangements. Other weaknesses were considered minor, such as a concern about self-selection bias in the discrete choice study, a need for more information on daily operations, and how information from other countries would be incorporated in the model. Overall, this is an application considered to be of high scientific impact.

DESCRIPTION (provided by applicant): Although several cervical cancer screening programs and demonstrations have taken place in southern Africa, only limited evaluations in the real-world setting have assessed program effectiveness, cost, and barriers/facilitators. Furthermore, even though numerous projects have been initiated, only a few successful programs are in operation today. Lessons learned from successful programs have not been synthesized and disseminated to foster scale-up of cost-effective implementation strategies. Given the substantial prevalence of human immunodeficiency virus (HIV) in southern Africa, which significantly increases the risk for cervical cancer, there is an urgent need to scale up prevention and screening services. The specific aims of the proposed research to address the gaps in the evidence base are as follows: Aim 1: Perform systematic process evaluation of the successful Cervical Cancer Prevention Program in Zambia (CCPPZ) to identify lessons for scaling up services in other sub-Saharan countries. Aim 2: Undertake comprehensive assessment of stakeholder perspectives on current program operations to identify barriers and facilitators and innovative financing arrangements to ensure sustainability. Aim 3: Develop a novel microsimulation model to assess cost and effectiveness of interventions and strategies for scaling up cervical cancer prevention and screening at the population level under real-world conditions that include budget and capacity constraints. Successful implementation of the project aims will provide evidence-based implementation guidelines on the optimal selection of prevention and screening interventions to reduce incidence of and mortality from cervical cancer. We will model the impact of behavior change (for example, sexual behavior affects rate of HIV and human papillomavirus [HPV] infections), prevention with HPV vaccination, and screening with visual inspection techniques and the new rapid HPV test; all scenarios will be evaluated under a variety of budget and capacity constraints. This comprehensive approach will allow policymakers to view the changes in cost and outcomes when interventions focusing on both HIV and cervical cancer are implemented rather than when a single-disease-focused approach is used. The process evaluation of CCPPZ and stakeholder assessment will provide best practices, key lessons, review of innovative financing options, and synthesis of barriers and facilitators to guide large-scale program implementation. To ensure generalizability of the findings to other sub-Saharan African nations, we will actively engage health professionals from African countries, and the findings will be disseminated and debated in two symposiums specifically convened for this purpose.

PUBLIC HEALTH RELEVANCE: Sub-Saharan Africa experiences an enormous burden from cervical cancer, but lessons learned from successful programs have not been synthesized and disseminated to

foster scale-up of cost-effective implementation strategies. To address this gap, we will perform a process evaluation of the successful prevention program in Zambia, undertake a comprehensive assessment of stakeholder perspectives and funding options, and develop an innovative model to assess cost and effectiveness under real-world conditions. This study will provide evidence-based implementation guidelines on the optimal selection of prevention and screening interventions that can be scaled up in sub-Saharan Africa to reduce mortality from cervical cancer.

CRITIQUE 1

Significance: 1
Investigator(s): 1
Innovation: 2
Approach: 2
Environment: 1

Overall Impact: This is a revised application that proposes to use a mixed methods approach to conduct extensive process evaluation of a successful cervical cancer prevention program in Zambia, obtain stakeholder perspectives on current operations, and ultimately develop a microsimulation model to assess cost and effectiveness of interventions and strategies for scaling up cervical cancer prevention and screening programs. The proposed study is highly significant in that it will provide evidence-based implementation guidelines on the optimal selection of prevention and screening interventions to reduce incidence of and mortality from cervical cancer in sub –Saharan African countries. It addresses a critical public health problem of risk for cervical cancer in southern Africa partly due to the prevalence of HIV infection. The proposed approach can have a substantial impact on policy makers deciding upon adoption or revision of a cervical cancer control program. Weaknesses include little detail about day to day operations and communication among various partners and limited discussion about how information gathered about other countries will be used; no mention on whether or not this information may inform modification or tailoring of the microsimulation model to better inform scale up efforts.

1. Significance:
Strengths
- If the aims of the study are accomplished, the resulting model and products will allow policymakers to view the changes in cost and outcomes when interventions focusing on both HIV and cervical cancer are implemented.
- The process evaluation of CCPPZ and stakeholder assessment will provide best practices, key lessons, review of innovative financing options, and synthesis of barriers and facilitators to guide large-scale program implementation.

Weaknesses
- There is limited information about how data obtained from stakeholders from other countries would be used to inform or further adapt the model to be more relevant for scale up in other countries.

2. Investigator(s):
Strengths
- The investigators are highly qualified with many years of relevant and successful research.
- Dr. Subamanian has extensive experience with large scale screening programs including those in low resources setting.
- The involvement of Dr. Sankaranarayanan from WHO IARC who directs the Screening and Early Detection Group is a strength of this proposal. He has extensive experience directing large randomized screening trials including the assessment of VIA in low resource settings.

- The investigators address concerns raised in the prior critique about roles of key personnel and these are appropriate.
- The team is well rounded, incudes individuals with expertise in areas relevant for the proposed study including process evaluation, community engagement, qualitative data analysis, and statistical techniques for analysis of quantitative data including mathematical modeling.

Weaknesses

- None noted.

3. Innovation:

Strengths

- Project is highly innovative in that it will be the first to use dynamic microsimulation to assess the process of acquiring HIV and HPV infections and the subsequent pathway to cervical cancer. The model then can be used to assess the value of programs that integrate prevention and screening options for HIV and HPV.
- The model will use data gathered through the first two aims and incorporate data from real-world implementation efforts to include parameters affecting scale up (such as access, quality, adherence, willingness to pay)

Weaknesses

- Methods used for process evaluation are not particularly innovative but seem appropriate.

4. Approach:

Strengths

- Uses a mixed methods evaluation protocol based on Re-AIM. Carries framework throughout; ties expected themes to be identified in the first two aims to framework.
- In response to reviewers comments, provide detailed description of procedures involved in the process evaluation (Aim 1) including both the qualitative and quantitative analyses; the approach is reasonable and will likely lead to data that will inform Aim 3 (the microsimulation model).
- Provides description of key measures and data sources for the process evaluation.
- Describes inputs for microsimulation model according to Re-AIM framework.

Weaknesses

- Little discussion about how the information obtained from the program database analysis (of cervical cancer screening programs in sub-Saharan Africa) and the face to face meetings with officials from other countries will be used to inform the microsimulation model or to modify it for use in other settings.

5. Environment:

Strengths

- Strong environment; research conducted at multiple sites with good coordination.
- UNC personnel based in Zambia will provide academic and research support and the Global Women's Health Fund—Zambia will provide administrative support and staff for data collection. Therefore, activities correspond with where expertise lies.
- Includes collaborations with the African Centre of Excellence for Women's Cancer Control (AfCoE), which coordinates the Cervical Cancer Prevention Program in Zambia and is co-directed by Dr. Parham
- Includes collaboration with the Mother and Child Health (MCDMCH), which oversees the clinics from which patients will be recruited for focus group and the discrete choice experiment.

Weaknesses

- None noted.

Protections for Human Subjects:
Acceptable Risks and/or Adequate Protections

- Adequately addressed concerns from prior review related to verbal consent, patient incentive, and data management and sharing.

Data and Safety Monitoring Plan (Applicable for Clinical Trials Only):
Acceptable

Inclusion of Women, Minorities and Children:

- Sex/Gender: Distribution justified scientifically
- Race/Ethnicity: Distribution justified scientifically
- Inclusion/Exclusion of Children under 21: Excluding ages < 21 justified scientifically
- Clinical practice guidelines recommend that women under the age of 25 should not be screened for cervical cancer.

Vertebrate Animals:
Not Applicable (No Vertebrate Animals)

Biohazards:
Not Applicable (No Biohazards)

Resubmission:

- Highly responsive to reviewer comments; adequately addressed issues related to details and roles of key investigators; clarified the link between HIV and HPV and underscored the innovative features of the proposed project related to that link; added detail about the process evaluation and the Discrete Choice Experiment; included description about future situation analyses using the model; added organizational chart to illustrate the relationships between collaborators.

Budget and Period of Support:
Recommend as Requested

CRITIQUE 2

Significance: 1
Investigator(s): 2
Innovation: 3
Approach: 3
Environment: 1

Overall Impact: This implementation research application seeks to learn lessons from a successful cervical cancer prevention program in Zambia, and use that information alongside local perceptions to develop a simulation model that will aid in planning and assessing scale up of such programs in other similar settings. The proposal is clear, and the methodological approach sound. The study aims take a logical approach from collecting the necessary information on key variables that would then be used in the model, followed by a test of the model. The investigator team is strong and has a proven track record of previous work in this area and context.

1. Significance:
Strengths

- This application seeks to learn lessons from the implementation of a successful cervical cancer prevention program in Zambia. If successful, it would yield very pertinent information on implementation and scaling up of such programs in other such settings.
- The modeling approach proposed in Aim 3 is a significant component that if successfully developed would be an important tool that can be used for simulating scale up of such programs in other settings.

Weaknesses

- None noted.

2. Investigator(s):

Strengths

- The PI has a track record of relevant research experience and the proposed application seems to fit in well with the PIs area of expertise
- There is a strong team of investigators with pertinent experience in cancer epidemiology and prevention, as well as modeling

Weaknesses

- None noted.

3. Innovation:

Strengths

- The proposed microsimulation modeling approach is a novel idea and will yield a useful tool for modeling scale up of such programs in a variety of different settings.

Weaknesses

- None noted.

4. Approach:

Strengths

- The project is built on a strong approach that first aims to collect information on implementation (Aim 1), and stakeholder perspectives (Aim 2), then use this information to develop a model that can be used to simulate different scenarios that may arise in the scale up of a cervical cancer prevention program.

Weaknesses

- None noted.

5. Environment:

Strengths

- RTI is well positioned to provide a supportive environment for the study. The availability of adequate IT support by the institution will be important for the success of this study.
- UNC and GWHF-Z have had presence in Zambia (infrastructure and personnel) that will be key to providing an enabling environment for this study

Weaknesses

- None noted.

Protections for Human Subjects:

Acceptable Risks and/or Adequate Protections

None noted

Data and Safety Monitoring Plan (Applicable for Clinical Trials Only):

Not Applicable (No Clinical Trials)

Inclusion of Women, Minorities and Children:

- Sex/Gender: Distribution justified scientifically

- Race/Ethnicity: Distribution not justified scientifically
- Inclusion/Exclusion of Children under 21: Excluding ages < 21 justified scientifically

Vertebrate Animals:
Not Applicable (No Vertebrate Animals)

Biohazards:
Not Applicable (No Biohazards)

Resubmission:
No concerns noted

Budget and Period of Support:
Recommend as Requested

CRITIQUE 3

Significance: 1
Investigator(s): 3
Innovation: 3
Approach: 5
Environment: 3

Overall Impact: The application proposes to develop evidence based implementation guidelines on the optimal selection of cervical cancer prevention and screening interventions, through performing a process evaluation on integrating HIV and cervical cancer prevention programs in Zambia, to reduce incidence of and mortality from cervical cancer in sub Saharan countries. If the project is successful, it will fill in the knowledge gap on cost effectiveness of the integrated programs in Zambia, which may inform policy makers in Zambia and other countries in sub Saharan Africa. The investigators are from various backgrounds, living in both the US and Zambia, and have lots of experiences in the proposed topics. The application proposes to develop a microsimulation model to assess cost and effectiveness of interventions under real world conditions, which is new to both research and policy makers in the sub Saharan Africa. The application will use mixed methods approach to process studies on costs and effectiveness (Aim 1), barriers or facilitators (Aim 2) of the program in Zambia, which will generate needed evidence for constructing the microsimulation model (Aim 3). To address the concerns raised in previous reviews, the application provides more details on data, analysis, modeling, and protecting human subjects. However, it still lacks sufficient details on developing innovative financing arrangements (user fee) to ensure sustainability. It is not clear whether the cost components include patient side costs. For data collected from the Discrete Choice Experiments, there is little discussion on voluntary participation and possible self-selection bias, and how that may affect the statistical results and developing microsimulation model. Overall, the application is likely to have medium impact.

1. Significance:
Strengths
If the project is successful,
- It will fill in the knowledge gap on cost-effectiveness of scaled up cervical cancer screening integrated with HIV interventions in Zambia, which may inform other countries in the sub-Saharan Africa.
- It will develop a micro-simulation model that enables other countries to assess the feasibility (based on budget constraints) of integrating and scaling up cervical cancer screening into HIV interventions.

- It will have important impact on public health by address preventing cervical cancer.

Weaknesses

- None noted.

2. Investigator(s):

Strengths

- The team is multidisciplinary with investigators from various backgrounds who have been working on the related topics.
- Some key investigators in the US are now working in Zambia and leading cervical cancer prevention program in the country.
- The PI has long-time collaborative experiences with some investigators.

Weaknesses

- Some Co-Investigators and local agencies (AfCoE and MCDMCH) will not be paid for their participation in the project, which raises concerns on how their timing and quality contributions will be guaranteed.

3. Innovation:

Strengths

- The application proposes assessing costs and effectiveness of integrating HIV and cervical cancer prevention in Zambia, which is not known in the region.
- The application will develop a microsimulation model to assess cost and effectiveness of interventions under real-world conditions that include budget and capacity constraints.

Weaknesses

- None noted.

4. Approach:

Strengths

- The proposal uses mixed methods to conduct process evaluation and undertake comprehensive assessment of stakeholder perspectives on current program operations to identify barriers and facilitators.
- The research will collect the data at various levels from stakeholders and also use the best available data to produce parameters for developing microsimulation model.
- Discrete choice experiments will be used to investigate patients/partners' preference over the screening programs.
- The application takes other African countries into consideration when developing microsimulation models.

Weaknesses

- The completion of Aim 3 will depend on the success of Aim1 and Aim 2: Aims 1 and 2 will provide data on implementation indicators that will be incorporated in the model to be developed in Aim 3. There are no discussions regarding the risks of processing Aims 1 and 2 and how that would affect processing Aim 3.
- The application proposes user-fee to sustain the scale-up of screening by assessing willingness to pay from patient-side. There is no sufficient information on (1) how to measure and analyze willingness to pay, (2) how to integrate the assessment of willingness into microsimulation, and (3) how to measure the tradeoff between user fees and patient compliance with screening.
- It is not clear whether the activity-based costs include patient-side costs such as transportation costs.

- For data collected from the Discrete Choice Experiments, there is little discussion on self-selection issue as a result of voluntary participation, and how self-selection may affect the statistical results and developing microsimulation model?
- Other concerns include: (1) microsimulation model will not be finalized and disseminated in the 2017 annual meetings; (2) references and text are not consistent.

5. Environment:
Strengths
- RTI and UNC have appropriate sources and facilities for the proposed project.
- UNC has a team in Zambia who will lead the local projects.

Weaknesses
- None noted.

Protections for Human Subjects:
Acceptable Risks and/or Adequate Protections
No concerns noted
Data and Safety Monitoring Plan (Applicable for Clinical Trials Only):
Not Applicable (No Clinical Trials)

Inclusion of Women, Minorities and Children:
- Sex/Gender: Distribution justified scientifically
- Race/Ethnicity: Distribution justified scientifically
- Inclusion/Exclusion of Children under 21: Excluding ages < 21 justified scientifically

Vertebrate Animals:
Not Applicable (No Vertebrate Animals)

Biohazards:
Not Applicable (No Biohazards)

Resubmission:
- To address the concerns raised in previous reviews, the application provides more details on data, analysis, modeling, and protecting human subjects. However, it still lacks sufficient details on developing innovative financing arrangements (user-fee) to ensure sustainability.

Resource Sharing Plans:
Acceptable

Budget and Period of Support:
Recommend as Requested

THE FOLLOWING SECTIONS WERE PREPARED BY THE SCIENTIFIC REVIEW OFFICER TO SUMMARIZE THE OUTCOME OF DISCUSSIONS OF THE REVIEW COMMITTEE, OR REVIEWERS' WRITTEN CRITIQUES, ON THE FOLLOWING ISSUES:

PROTECTION OF HUMAN SUBJECTS (Resume): ACCEPTABLE

INCLUSION OF WOMEN PLAN (Resume): ACCEPTABLE

INCLUSION OF MINORITIES PLAN (Resume): ACCEPTABLE

INCLUSION OF CHILDREN PLAN (Resume): ACCEPTABLE

COMMITTEE BUDGET RECOMMENDATIONS: The budget was recommended as requested.

NIH has modified its policy regarding the receipt of resubmissions (amended applications). See Guide Notice NOT-OD-14-074 at http://grants.nih.gov/grants/guide/notice-files/NOT-OD-14-074.html. The impact/priority score is calculated after discussion of an application by averaging the overall scores (1-9) given by all voting reviewers on the committee and multiplying by 10. The criterion scores are submitted prior to the meeting by the individual reviewers assigned to an application, and are not discussed specifically at the review meeting or calculated into the overall impact score. Some applications also receive a percentile ranking. For details on the review process, see http://grants.nih.gov/grants/peer_review_process.htm#scoring.

MEETING ROSTER

Dissemination and Implementation Research in Health Study Section
Healthcare Delivery and Methodologies Integrated Review Group
CENTER FOR SCIENTIFIC REVIEW
DIRH
October 14, 2015 - October 15, 2015

CHAIRPERSON

GUYDISH, JOSEPH R, MPH, PHD
PROFESSOR IN RESIDENCE, MEDICINE AND PSYCHIATRY
INSTITUTE FOR HEALTH POLICY STUDIES
SCHOOL OF MEDICINE
UNIVERSITY OF CALIFORNIA, SAN FRANCISCO
SAN FRANCISCO, CA 94143

MEMBERS

BACHANI, ABDULGAFOOR M *
ASSISTANT PROFESSOR
INTERNATIONAL HEALTH
HEALTH SYSTEMS
JOHNS HOPKINS UNIVERSITY
BALTIMORE, MD 21205

BENZO, ROBERTO PABLO, MD *
ASSOCIATE PROFESSOR
DIVISION OF PULMONARY & CRITICAL CARE
MAYO CLINIC COLLEGE OF MEDICINE
ROCHESTER, MN 55902

CARRASQUILLO, OLVEEN , MPH, MD
PROFESSOR OF MEDICINE AND PUBLIC HEALTH SCIENCES
CHIEF, DIVISION OF INTERNAL MEDICINE
DIVISION HEAD, HEALTH SERVICES RESEARCH AND POLICY
MILLER SCHOOL OF MEDICINE
UNIVERSITY OF MIAMI
MIAMI, FL 33101

DULIN, MICHAEL F, MD, PHD *
CHIEF CLINICAL OFFICER
DEPARTMENT OF FAMILY MEDICINE
CAROLINAS MEDICAL CENTER
CHARLOTTE, NC 28212

ELLERBECK, EDWARD F, MPH, MD
PROFESSOR
DEPARTMENT OF PREVENTIVE MEDICINE
UNIVERSITY OF KANSAS MEDICAL CENTER
KANSAS CITY, KS 661607313

FERNANDEZ, MARIA E, PHD
PROFESSOR OF HEALTH PROMOTION AND BEHAVIORAL SCIENCES
SCHOOL OF PUBLIC HEALTH
UNIVERSITY OF TEXAS HEALTH SCIENCE CENTER AT HOUSTON
HOUSTON, TX 77030

FRITZ, JULIE M, PHD
PROFESSOR
ASSOCIATE DEAN FOR RESEARCH
COLLEGE OF HEALTH
UNIVERSITY OF UTAH
SALT LAKE CITY, UT 84102

HAMMETT, THEODORE M, PHD *
VICE PRESIDENT AND PRINCIPAL ASSOCIATE
INTERNATIONAL HEALTH DIVISION
ABT ASSOCIATES INCORPORATED
CAMBRIDGE, MA 02138

HOUSTON, THOMAS K II, MPH, MD
PROFESSOR
DEPARTMENT OF QUANTITATIVE HEALTH SCIENCES
CHIEF, DIVISION OF HEALTH INFORMATICS AND IMPLEMENTATION SCIENCE
UNIVERSITY OF MASSACHUSETTS MEDICAL SCHOOL
WORCESTER, MA 01655

HUDSON, SHAWNA V, PHD
ASSOCIATE PROFESSOR AND ASSOCIATE DIRECTOR FOR RESEARCH
DEPARTMENT OF FAMILY MEDICINE AND COMMUNITY HEALTH
ROBERT WOOD JOHNSON MEDICAL SCHOOL
RUTGERS UNIVERSITY
SOMERSET, NJ 08873

LEWIS, CARA CHARISSA, PHD *
BECK SCHOLAR, ASSISTANT PROFESSOR
DEPARTMENT OF PSYCHOLOGICAL AND BRAIN SCIENCES
INDIANA UNIVERSITY
BLOOMINGTON, IN 47405

LU, CHUNLING , PHD *
ASSISTANT PROFESSOR
DEPARTMENT OF GLOBAL HEALTH AND SOCIAL MEDICINE
HARVARD MEDICAL SCHOOL
HARVARD UNIVERSITY
BOSTON, MA 02115

MAYS, GLEN P, PHD *
PROFESSOR
HEALTH SERVICES AND SYSTEMS RESEARCH
COLLEGE OF PUBLIC HEALTH
UNIVERSITY OF KENTUCKY
LEXINGTON , KY 40536

MCLEOD, BRYCE DOUGLAS, PHD *
ASSOCIATE PROFESSOR
DEPARTMENT OF PSYCHOLOGY
VIRGINIA COMMONWEALTH UNIVERSITY
RICHMOND , VA 23284

SALDANA, LISA , PHD
SENIOR SCIENTIST
OREGON SOCIAL LEARNING CENTER
EUGENE, OR 97401

SHELLEY, DONNA R, MPH, MD
ASSOCIATE PROFESSOR
VICE CHAIR OF RESEARCH DEVELOPMENT
SCHOOL OF MEDICINE
NEW YORK UNIVERSITY
NEW YORK, NY 10016

SHI, LIZHENG , PHD *
REGENTS ASSOCIATE PROFESSOR
DEPARTMENT OF GLOBAL HEALTH SYSTEMS
AND DEVELOPMENT
TULANE UNIVERSITY SCHOOL OF PUBLIC HEALTH AND
TROPICAL MEDICINE
NEW ORLEANS, LA 70112

SIMON, MELISSA ANDREA, MPH, MD, PHD
GEORGE H. GARDNER ENDOWED ASSOCIATE
PROFESSOR
VICE CHAIR OF CLINICAL RESEARCH
DEPARTMENT OF OBSTETRICS AND GYNECOLOGY
PREVENTIVE MEDICINE AND MEDICAL SOCIAL
SCIENCES
NORTHWESTERN UNIVERSITY
CHICAGO, IL 60611

WILLIAMS, OLAJIDE , MD
ASSOCIATE PROFESSOR OF CLINICAL NEUROLOGY
DEPARTMENT OF CLINICAL NEUROLOGY
COLUMBIA UNIVERSITY MEDICAL CENTER
NEW YORK, NY 10032

WILSON, MARK G *
PROFESSOR
ASSOCIATE DEAN FOR ACADEMIC AFFAIRS
UGA FOUNDATION PROFESSOR IN PUBLIC HEALTH
DEPARTMENT OF HEALTH PROMOTION AND BEHAVIOR
UNIVERSITY OF GEORGIA
ATHENS, GA 30602

WINICKOFF, JONATHAN P, MPH, MD
ASSOCIATE PROFESSOR OF PEDIATRICS
DEPARTMENT OF PEDIATRICS
HARVARD MEDICAL SCHOOL
BOSTON, MA 02114

MAIL REVIEWER(S)

GABRIELSON, EDWARD W., MD
PROFESSOR
DEPARTMENT OF PATHOLOGY
JOHNS HOPKINS CANCER CENTER
BALTIMORE, MD 21231

GOFFMAN, LISA , PHD
PROFESSOR
DEPARTMENT OF AUDIOLOGY AND SPEECH SCIENCES
PURDUE UNIVERSITY
WEST LAFAYETTE, IN 47907

NING, JING , PHD
ASSISTANT PROFESSOR
DEPARTMENT OF BIOSTATISTICS
DIVISION OF QUANTITATIVE SCIENCES
UNIVERSITY OF TEXAS MD ANDERSON CANCER CENTER
HOUSTON, TX 77030

SCIENTIFIC REVIEW OFFICER

HARE, MARTHA L, PHD
SCIENTIFIC REVIEW OFFICER
CENTER FOR SCIENTIFIC REVIEW
NATIONAL INSTITUTES OF HEALTH
BETHESDA, MD 20892

EXTRAMURAL SUPPORT ASSISTANT

BARTLETT, VALERIE
EXTRAMURAL SUPPORT ASSISTANT
CENTER FOR SCIENTIFIC REVIEW
NATIONAL INSTITUTES OF HEALTH
BETHESDA, MD 20892

OTHER REVIEW STAFF

FOSU, GABRIEL B, PHD
SCIENTIFIC REVIEW OFFICER
CENTER FOR SCIENTIFIC REVIEW
NATIONAL INSTITUTES OF HEALTH
BETHESDA, MD 20892

* Temporary Member. For grant applications, temporary members may participate in the entire meeting or may review only selected applications as needed.

Consultants are required to absent themselves from the room during the review of any application if their presence would constitute or appear to constitute a conflict of interest.
